# A Review of Factors Influencing the National Board of Medical Examiners Clinical Subject Examination (NBME) Neurology Subject Scores

**DOI:** 10.7759/cureus.86165

**Published:** 2025-06-16

**Authors:** Mariam Khalil, Samuel Salib, Meghana Renavikar, Austin Lee, Anand Dhaliwal, Vijay Khatri

**Affiliations:** 1 Neurology, California Northstate University College of Medicine, Elk Grove, USA; 2 Otolaryngology - Head and Neck Surgery, California Northstate University College of Medicine, Elk Grove, USA; 3 Surgery, California Northstate University College of Medicine, Elk Grove, USA; 4 Surgical Oncology, California Northstate University College of Medicine, Elk Grove, USA

**Keywords:** medical student, medical student clerkship, nbme shelf, nbme subject exam, neurology

## Abstract

The National Board of Medical Examiners Clinical Subject Examination (NBME) Neurology subject exam scores of third- and fourth-year medical students are a critical objective component of their application for a neurology residency. Multiple factors, including scheduling, preclinical preparation, and educational methods, may influence a student's performance on the Neurology NBME Clinical Science Exam (Shelf exam) during their clerkship. This review was conducted following the Preferred Reporting Items for Systematic Reviews and Meta-Analyses (PRISMA) guidelines to identify factors that impact medical students' performance on the NBME Neurology subject exam during their clerkship. This review synthesizes existing research to identify evidence-based strategies that optimize student performance on the Neurology Shelf exams. Understanding the factors influencing NBME scores is essential for medical students and clerkship directors to enhance educational outcomes and residency preparedness. The findings of this systematic review highlight the importance of scheduling, preclinical factors, and variations in educational methods in determining students' performance on the Neurology Shelf exam.

## Introduction and background

To accurately assess medical knowledge acquired during clerkships, the National Board of Medical Examiners Clinical Subject Examination (NBME) Clinical Science exam is used to objectively evaluate students in US Liaison Committee on Medical Education-accredited medical schools [1]. The exam is an objective measurement of student medical knowledge, unlike subjective measures such as Objective Structured Clinical Examination (OSCE) results, clerkship examinations, and evaluation by preceptors during the neurology rotation. The NBME Subject Examination in Clinical Neurology, colloquially known as the Neurology Shelf exam, is given to assess student knowledge of a specific discipline, clinical neurology, at the end of the clerkship rotation [1].

Shelf exams are scored using an equated percentage system ranging from 0 to 100%, with institutional variations in their use for grading [1]. Shelf exams may be used as a final exam, extra credit, a pass-fail test, or a determinant of honors [2]. As of 2020, the Association of American Medical Colleges (AAMC) reports that 84% of medical schools have neurology as a mandatory clerkship [3]. Although shelf scores are not directly reported to residency programs, they are an integral component of the final grade the student receives for the neurology clerkship. Residency programs value honors in clinical rotations as a key factor when selecting candidates for interviews [4]. Additionally, given that the majority of US medical schools include neurology as a core rotation, strong performance on the neurology NBME exam is imperative for increasing a student’s ranking among peers at their home institution [3]. In the past, neurology directors evaluated the United States Medical Licensing Examination (USMLE) Step 1 and 2 scores, in addition to holistic aspects of the applicant, such as geographic preference and interpersonal skills, to rank residency candidates [5]. Step 1 has now become pass/fail, and hence there is more reliance on the Step 2 score in addition to the grade achieved for the specific M3 clerkship.

There is limited comprehensive research identifying the specific factors that influence medical students' performance on the Neurology Shelf exam. By addressing this knowledge gap, the review aims to provide valuable insight to clerkship directors and educators, enabling them to design and implement effective medical education programs that optimize students' learning and performance in neurology. Our findings emphasize the importance of considering the factors that both positively and negatively impact NBME Shelf scores. Given that there are innumerable factors outside of the traditional classroom that may influence a student’s shelf exam performance, this review emphasized determining what institution-related and resource availability-related factors may be implemented to strengthen a student’s Neurology Shelf exam score. By gaining insights into these factors, medical schools can make informed decisions about curriculum design, resource allocation, and support systems to enhance students' learning experiences and improve their performance on the NBME Neurology subject exam.

## Review

Methods

**Figure 1:**
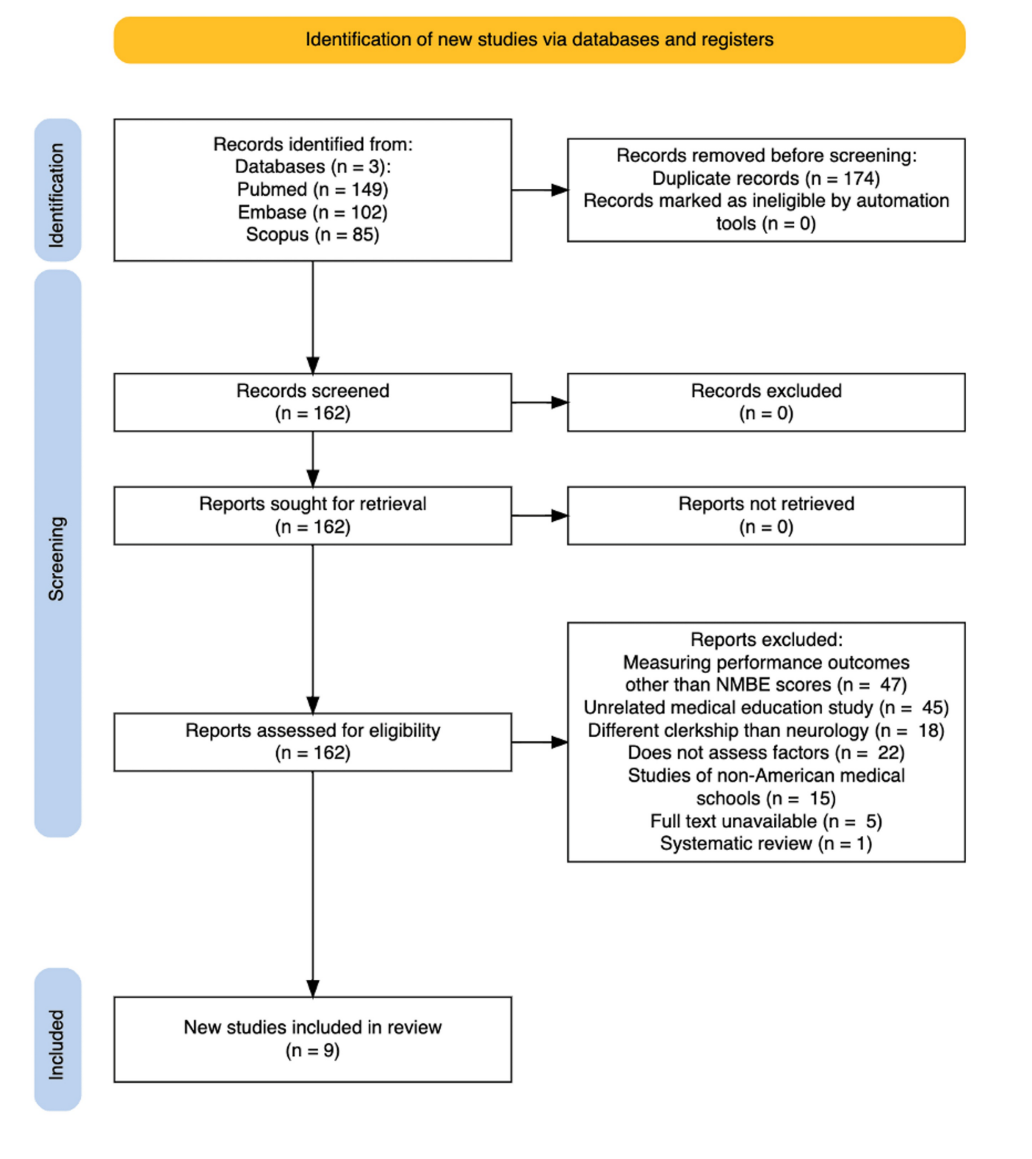
Article selection process PRISMA: Preferred Reporting Items for Systematic Reviews and Meta-Analyses

Two authors independently conducted a comprehensive literature search in Embase, Scopus, and PubMed on November 14, 2023, adhering to the Preferred Reporting Items for Systematic Reviews and Meta-Analyses (PRISMA) guidelines. The search strategy included the keywords "student" and "clerkship" and “neurology” and is limited to studies in English with full text available, which were published within the past 10 years. Duplicate records were removed using Rayyan reference management software (Rayyan Systems Inc., Cambridge, MA) manually, and the abstracts of the remaining records were screened for relevance by two authors independently. A third author was consulted when an agreement could not be reached to eliminate bias in the screening process. A full-text assessment was done of 336 studies, out of which 174 duplicates and 153 articles were excluded due to failure to meet inclusion criteria. The nine remaining studies were included in this systematic review. Data on the factors influencing subject exam scores were independently extracted by three authors and synthesized in a Word document. Figure 1 illustrates the article selection process, following PRISMA guidelines, that was used in this study.

Inclusion criteria for the review included studies that had a comparison of factors that impact student performance on the NBME Neurology subject exam. Exclusion criteria included studies that did not have full text available, did not assess any factors, did not report neurology subject exam scores or p-values, and were not specific to neurology, other systematic reviews, and papers reporting on non-American medical schools. The final list of articles included in the review consisted of nine articles, which met our inclusion criteria. The selected articles were reviewed, and the relevant data were extracted and organized into tables. The main factors pertinent to this research were identified by grouping them into broad categories, derived from recurring themes found in the data. Given the heterogeneity of study designs and outcomes, a narrative synthesis was used for data analysis.

Results

The search retrieved 336 articles: 102 from Embase, 85 from Scopus, and 149 from PubMed. After screening, 162 were excluded, and nine met the PRISMA criteria for inclusion (Figure 1). The nine articles were classified into three primary groupings based on the focal points of the factors they highlighted. The first category included articles that examined preclinical factors. The second grouping comprised articles that examined the timing of the neurology clerkship relative to other clerkships, for variables such as scheduling and duration. The final category consisted of papers examining educational method types during the clerkship. Studies addressing multiple influencing factors were included in more than one table where applicable. Finally, all the relevant factors, results, and P-values were organized into tables.

Preclinical Factors 

The impact of preclinical factors, such as education, mindset, and resources, on NBME Neurology subject exam scores was determined. These articles addressed the current status of medical education and how curriculum changes may impact future academic performance (Table 1). Strowd et al. [6] found that third-year medical students performed better on their neurology clerkships by setting pre-determined neurology-specific goals through the influence of advisors and peers. The students in the goal cohort completed a baseline survey at the time of clerkship, self-generating a list of their top 3 goals for the four-week clerkship. Achievers of these goals had NBME scores 1.7 points higher than their non-achiever counterparts (95% confidence interval: 0.1-3.2, p = 0.04) who did not set predetermined goals [6]. Haidar et al. [7] studied the incorporation of a longitudinal ultrasound curriculum that focused on head and neck, cardiovascular, abdominal, musculoskeletal, and procedural ultrasound sessions during the 1.5-year preclinical curriculum divided into blocks by organ system. The organ systems were divided into cardiovascular, pulmonary, renal, gastrointestinal, hematology/oncology, endocrine/reproduction, musculoskeletal, neurology, dermatology, psychiatry, and infectious diseases blocks. There was no significant difference in preclinical neurology block, NBME Neurology Shelf exam scores (p = 0.212), and Step 1 scores for the ultrasound group compared to those without the ultrasound curriculum [7].

**Table 1:** Preclinical factors and their effect on NBME neurology subject exam scores NBME: National Board of Medical Examiners Clinical Subject Examination

Author (year)	Variable Assessed	Shelf Score Result (SD)	NBME Shelf Score Correlation, r (95% confidence interval)	P-value
Strowd et al. (2016) [6]	Goal achievers	77.7 (7.4)	Not provided	0.2
	Goal non-achievers	75.7 (7.2)		
Haidar et al. (2022) [7]	Ultrasound curriculum	87.1 (6.0)	Not provided	0.212
	No ultrasound curriculum	86.4 (6.5)		

Educational Method 

Next, the impact of various educational resources, such as case volume, OSCE exams, and outpatient rotation opportunities, on NMBE Neurology subject exam scores was examined (Table 2). Albert et al. [8] found that the volume of cases a medical student encountered was significantly correlated with higher performance on measures of specialty knowledge and clinical skill. Thereby, an increased case volume significantly improved both the neurology NBME score(r = 0.0.142, p = 0.005) and the OSCE physical examination performance scores (r = 0.136, p = 0.007) [8]. Two years later, the study was repeated, and Albert et al. [9] found that the volume of cases a medical student encountered was significantly correlated with the Neurology NBME (r = 0.290, p < 0.001), the OSCE physical examination (r = 0.236, p = 0.011), and the OSCE patient clinical note (r = 0.238, p = 0.010) scores. In addition, they examined whether the breadth of the neurology cases observed by the student classified into 13 categories (seizure, cerebrovascular disease, peripheral/neuromuscular, spinal disease/neck/back pain, central nervous system demyelinating disease/neuro-immunology, headaches, sleep disorders, neuro-oncology, movement disorders, dementia, other neurodegenerative disorders) influenced objective scores. They found that the breadth of neurology cases significantly correlated with the Neurology NBME scores (r = 0.231, p = 0.017), though a significant correlation was noted with the OSCE score [9]. Sampat et al. [10] found that having students complete a two-week outpatient rotation in which they worked with a single general neurologist, instead of multiple different subspecialists, did not significantly affect shelf examination (p = 0.59) or standardized evaluation (p = 0.34) scores. However, the study found that taking a neurology shelf pre-test correlated with overall higher standardized evaluation scores (p < 0.01) and higher shelf examination scores (p < 0.01) [10]. Thompson Stone et al. [11] reported that incorporating a formal bedside skills evaluation (BSE) for third-year students resulted in an overall positive correlation with their shelf scores of 0.35 (p < 0.001). Lukas et al. [12] found that students who performed better in their OSCE, as indicated by their OSCE scores, did better on the NBME Neurology scores (R2Δ = 0.131, p < 0.001) and received better faculty clinical scores (R2Δ = 0.078, p < 0.001.

**Table 2:** Educational methods and their effect on NBME Neurology subject exam scores NBME: National Board of Medical Examiners Clinical Subject Examination

Author (year)	Variable Assessed	Shelf Score Result (SD)	NBME Shelf Score Correlation (r)	P-value
Albert et al., (2014) [8]	Volume of patients and correlation to NBME Neurology score	Not reported	1) r = 0.142	1) p = 0.005
Albert et al. (2016) [9]	Volume of patients and correlation to NBME Neurology score	Not reported	r = 0.290	p < 0.01
Sampat et al. (2019) [10]	1) No practice exam	1) 78.63 (7.13)	Not reported	1 vs 2: <0.01
	2) NeuroSAE practice exam	2) 80.66 (7.17)		1 vs 3: <0.001
	3) NBME practice exam	3) 84.10 (5.04)		4 vs 5: 0.59
	4) Rotation with specialist	4) 80.59 (7.36)		
	5) Rotation with general neurologist	5)80.17 (6.83)		
Albert et al. (2016) [9]	1) OSCE category: diagnostic evaluation	Not reported	1) r = -0.059	0.976
	2) OSCE category: therapeutic intervention		2) r = 5.4	0.000001
	3) OSCE category: supportive intervention		3) r = 2.1	0.017
Thompson Stone et al. (2016) [11]	Bedside skills evaluation (BSE) year 1	79.3 (7.7)	Not provided	p < 0.0001
	Bedside skills evaluation (BSE) year 2	78.2 (7.8)		
Lukas et al. (2014) [12]	OSCE Score	Not reported	R2 = 0.131	p < 0.001

Clerkship Structure 

Finally, the relevance of the timing of a medical student's neurology clerkship, in comparison to other clerkships during their third and fourth years, was examined (Table 3). Sampat et al. found that, for every additional core clerkship that a student completed, regardless of which clerkship it was, there was an increase of 0.72 points in the Neurology NMBE subject exam score 1 [10]. Monrad et al. [13] examined the transition period between curricular changes where the clerkship education was reduced from 48 weeks to 36 weeks, the neurology clerkship was reduced from four weeks to three weeks of mixed inpatient/outpatient experiences with a reduction in didactic sessions, and the neuro-ophthalmology conference. They found that, when the neurology clerkship length was shortened by 25%, there was no significant difference in NBME subject exam scores between the cohort with the shortened clerkship and the control group [13]. Nackers et al. [14] also observed a change when they converted their curriculum from a traditional 2+2 to an integrated clinical education. The traditional neurology clerkship was a four-week block, whereas, in the new curriculum, two weeks of neurology were integrated in the “acute care module” (internal medicine, emergency medicine, neurology, and psychiatry) and then another two weeks in the “chronic and preventative care module” (family medicine, general internal medicine, neurology, and psychiatry). However, for both curricula, the neurology shelf exam was taken after completion of the full four weeks of clinical rotation. There was no statistical difference in scores between having an integrated curriculum in which students begin clinical rotations one semester earlier during their second year and the traditional 2+2 medical school educational model in which a student completes two years of preclinical education, followed by two years of clinical education, with an integration of the traditional clerkships into four blocks [14,15].

**Table 3:** Clerkship structures and their effect on NBME Neurology subject exam scores NBME: National Board of Medical Examiners Clinical Subject Examination

Author (year)	Variable Assessed	Shelf Score Result (SD)	Shelf Score Correlation, r ( 95% confidence interval)	P-value
Sampat et al. (2019) [10]	1) Surgery clerkship taken before neurology	81.77 (6.91)	Not provided	1 vs 2: < 0.01
	2) Neurology clerkship taken before surgery	79.22 ± 6.98		1 vs 2: < 0.01
	3) Clerkship completed prior to neurology	3) +0.72 (.27) Per clerkship taken before neurology		3) 0.007
Monrad et al. (2018) [13]	25% shorter clerkship (4.5 weeks)	1) 86.2 (5.2)	Not provided	0.015
	Traditional length (6 weeks)	2) 87.2 (5.6)		
Nackers et al. (2022) [14]	Integrated curriculum (ForWard)	77.1 (75.3-78.9)	0.52 (from -1.31 to 2.36)	p > 0.005
	Traditional curriculum	77.7 (75.7-79.6)		

Discussion

This systematic review highlights the complex interplay of factors influencing success in neurology clinical education. While certain factors may appear intuitively significant, detailed analysis reveals complex relationships and opportunities for targeted educational improvements. This review explores three key categories: pre-clerkship preparation, clerkship structure, and educational methods.

While some findings might seem counterintuitive, they provide valuable information for optimizing educational strategies. For example, shortening the clerkship by 25% did not significantly affect score outcomes [11]. Additionally, factors related to the preclinical years, such as setting goals or participating in an ultrasound curriculum before the clerkship, did not show a significant impact on exam performance [13]. Interestingly, the timing of the neurology clerkship played a crucial role in student scoring, with prior exposure to other core clerkships positively correlating with higher scores. The timing of the neurology clerkship significantly influenced student scores, with prior exposure to other core clerkships correlating with higher performance [11]. Resource availability for medical students emerged as another important factor. Busy rotation sites, access to practice exams, and strong faculty mentorship (both specialists and general neurologists) all correlated with better performance [10].

Although factors preceding clinical rotations, such as goal setting, mindset, and resource accessibility, did not demonstrate a statistically significant influence on NBME Neurology Shelf exam scores, they could still play a crucial role in fostering interest and motivation for the field. Studies suggest that students who set neurology-specific goals and hold a positive view of the subject tend to perform better, potentially hinting at the link between early engagement and future career paths [6]. This raises intriguing questions about whether interventions designed to spark a passion for neurology during preclinical years could ultimately help address the field's growing need for specialists. Seven factors were found to influence a medical student’s interest in a specific specialty prior to rotations, including medical lifestyle, encouragement, positive clinical exposure, discouragement or negative clinical exposure, economics or politics, competence or skills, and ease of residency entry [16]. Therefore, optimizing NBME performance and clinical clerkship experience could help cultivate medical students' interest in neurology.

A variety of educational exposures significantly impacted NBME Neurology Shelf exam scores, but their ultimate effects varied. The hands-on clinical experience proved beneficial, with studies showing a weakly positive correlation between case volume and breadth and shelf exam scores [8,9]. This suggests prioritizing busier rotation sites to obtain maximal exposure of case variety. Additionally, strong performance in subjective assessments such as OSCE and faculty clinical scores translated to higher exam scores [11]. Apart from OSCE examinations, taking a neurology shelf practice exam, including both the NeuroSAE practice exam and NBME practice exam, showed statistically significant improvement in the actual NBME neurology exam [10].This highlights that offering more study resources for students, even in clinical rotation, leads to improved performance on exams. Sidhu et al. [16] found that, when students were offered a comprehensive review prior to their NBME Psychiatry Subject Exam (PSE), they performed significantly better, with the average score being 84.53 versus 77.15 for matched controls (p < 0.00010). This knowledge has the potential to be applied to neurology education to benefit student performance. Finally, there was no statistically significant difference between a rotation done with a general neurologist versus a specialized neurologist [1]. However, this does not negate some of the valuable education that students may gain from doing a rotation with a specialist neurologist, which may not result in a higher exam score.

Additionally, student differences in OSCE style, as noted by scorers, held predictive value for the Neurology NBME exam, showcasing the complex interplay of factors influencing performance [17]. OSCE styles were defined as supportive, therapeutic, and diagnostic. Afterwards, students were scored in these three different categories, and the predictive power of their score on the NBME neurology subject exam was assessed. OSCE scores only in the therapeutic and supportive OSCE categories correlated significantly with both NBME written exam scores and composite faculty clinical scores, suggesting that OSCE performance reflects not only specific neurology skills but also broader clinical competency. This relationship between OSCE style and NBME Neurology scores may also inspire clerkship directors to place more emphasis and share learning resources on the therapeutic and supportive aspects of the neurology clerkship. Overall, faculty clinical evaluations across different rotations lack consistent correlation, highlighting potential subjectivity, yet NBME standardized exams offer a reliable and objective measure of student performance. These findings support the use of OSCE during preclinical years only as a precursor to a strong performance in the neurology clerkship and suggest OSCE performance as a valuable tool for informing both student progress and educational program development. Furthermore, analyzing OSCE scores from two standardized patient encounters revealed high internal consistency and predictive validity; however, the study noted the shortcomings of this approach when it comes to external validity and generalizability, as this is a subjective testing measure [12].

Clerkship structure emerged as a key factor influencing exam performance. Completing more clerkships before neurology boosted scores [10]. Intriguingly, other studies on clerkship order in internal medicine and surgery clerkships suggest that sequence can impact performance on their respective shelf exam [10]. One potential explanation for this phenomenon may be that prior exposure to relevant knowledge from other fields, such as neuroanatomy in anatomy courses or neuro-pathology in internal medicine clerkships, might benefit students during a subsequent neurology clerkship. This prior knowledge could then serve as a foundation for building deeper neurology understanding and potentially even increasing exam scores. Additionally, clerkship structure modification, such as the implementation of an integrated curriculum (e.g., the "ForWard" model), showed no significant difference compared to traditional curricula [15]. The ForWard curriculum condenses preclinical studies into just three phases, with the second phase focusing on "types of care," instead of individual specialties. Importantly, both curricula require students to take the same board exams [14]. This finding highlights that using these integrated curricula may lead to more positive outcomes without compromising the core education of medical students. Shortening the neurology clerkship length from six to 4.5 weeks had no statistically significant impact on exam scores, suggesting that a shorter curriculum might not be detrimental to student performance in neurology and that time may be repurposed for other rotations that may involve a more expansive variety of cases [18]. It was found that a longer Internal Medicine clerkship resulted in higher test scores (coefficient = 0.23 points/week; p-value < 0.01) [18]. Thus, it may be valuable for clerkship directors to shorten the length of neurology clerkships and repurpose that time to internal medicine. Ultimately, the structure of the clerkship is crucial not only for test scores but ensuring that medical students are not compromising on any part of their education.

Despite the strengths of this systematic review, several limitations must be acknowledged. A major concern is the heterogeneity of study designs - many were retrospective, relied on institutional or self-reported data, and lacked randomization or control groups, introducing potential recall and selection biases and limiting causal interpretation. There was also significant variation in outcome measures, with studies reporting NBME performance as mean scores, percentiles, or correlations. Some focused on curriculum changes, while others examined subjective factors such as student mindset. These inconsistencies reduce the comparability and accuracy of aggregated findings. Furthermore, although prior research has explored student-patient interactions, a critical gap remains in understanding how educational environments and clerkship structures influence outcomes. Comparing inpatient and outpatient experiences across disciplines may help optimize resource use and enhance student learning.

Future research should address these limitations by incorporating a wider range of medical schools and considering additional factors potentially impacting shelf exam scores. Such comprehensive investigations can build upon our findings and refine our understanding of how to foster success in neurology education.

## Conclusions

An assessment of numerous studies demonstrated that three main positive factors influenced the Neurology Shelf Exam scores of third-year medical students. Based on the findings, medical schools and educators are encouraged to implement several targeted strategies. First, increasing preclinical exposure to neurology, such as through integrated neuroscience modules, early clinical shadowing opportunities, or neurology-focused case discussions, can help cultivate interest and foundational knowledge. Second, ensuring the timely scheduling of the neurology clerkship - ideally after students have completed core rotations like internal medicine - may allow students to apply broader clinical reasoning skills to neurology cases, thereby improving performance. Third, institutions should prioritize equitable access to high-quality educational resources, including NBME-aligned practice materials, neurology review texts, and digital question banks. Faculty-led review sessions and mentorship programs can further enhance students' preparedness.

Beyond improving test scores, these interventions may elevate the overall quality of neurology education, helping foster greater student confidence, deeper clinical understanding, and potentially increased interest in neurology as a career. By aligning clerkship structure and resource distribution with evidence-based practices, medical schools can better prepare students not only for assessments but for competent, compassionate care of patients with neurological conditions. This is the first systematic review of factors influencing Neurology NBME scores, providing insights for curriculum optimization and educational quality assessment.

## Appendices

**Table 4:** Exclusion criteria table

Title	Year	Authors	Exclusion Reason
An Objective Structured Clinical Exam on Breaking Bad News for Clerkship Students: In-Person Versus Remote Standardized Patient Approach	2023	Prasad	Different clerkship from neurology
Impact of Students' Scheduling Choice on Clerkship Examination Score Performance in a Time-Varying Competency-Based Curriculum	2023	Kraakevik	Different clerkship from neurology
Pre-clerkship Teaching and Learning in the Virtual Learning Environment: Lessons Learned and Future Directions	2022	Sewell	Different clerkship from neurology
Prematriculation Healthcare Employment Predicts Success in Clerkship Environment	2020	Strowd	Different clerkship from neurology
Independent or Integrated? The Impact on Subject Examination Scores of Changing a Neuropsychiatry Clerkship to Independent Clerkships in Psychiatry and Neurology	2017	Anderson	Different clerkship from neurology
Predictors of medical school clerkship performance: a multispecialty longitudinal analysis of standardized examination scores and clinical assessments	2016	Casey	Different clerkship from neurology
Improving medical student recruitment into neurological surgery: a single institution's experience	2013	Agarwal	Different clerkship from neurology
Preclinical Surgical Preparatory Course and the NRMP Match: Early Exposure and Surgical Recruitment a 10-Year Follow-Up	2020	Anderson	Different clerkship from neurology
Impact of a Revised Curriculum Focusing on Clinical Neurology and Musculoskeletal Care on a Required Fourth-Year Medical Student Physical Medicine and Rehabilitation Clerkship	2016	Norbury	Different clerkship from neurology
Medical Student Attitudes and Perceptions on the Relevance of Neuroscience to Psychiatry: A Mixed Methods Study	2022	Porter-Stransky	Different clerkship from neurology
Teaching scripts via smartphone app facilitate resident-led teaching of medical students	2021	Zessis	Different clerkship from neurology
A Required, Combined Neurology-Physical Medicine and Rehabilitation Clerkship Addresses Clinical and Health Systems Knowledge Gaps for Fourth-Year Medical Students	2021	Curtis	Different clerkship from neurology
The Successful Integration of Psychiatry and Neurology in a Combined Clerkship	2017	Griffeth	Different clerkship from neurology
Are Scores From NBME Subject Examinations Valid Measures of Knowledge Acquired During Clinical Clerkships?	2017	Ryan	Different clerkship from neurology
Simulation-Based Mastery Learning Improves Medical Student Performance and Retention of Core Clinical Skills	2016	Reed	Different clerkship from neurology
Impact of a structured review session on medical student psychiatry subject examination performance	2015	Siddiqi	Different clerkship from neurology
Redesign and implementation of the radiology clerkship: From traditional to longitudinal and integrative	2014	Di Salvo	Different clerkship from neurology
Creating a longitudinal integrated clerkship with mutual benefits for an academic medical center and a community health system	2014	Poncelet	Different clerkship from neurology
A Call to Action: Using Curriculum Mapping at Four Medical Schools in Massachusetts to Advance Serious Illness Communication Training in Undergraduate Medical Education	2023	Reidy	Does not asses factors
Creating ophthalmology experiences in undergraduate medical education: pilot of a case-based learning ophthalmology tool	2023	Tran	Does not asses factors
Essential anatomy for core clerkships: A clinical perspective	2023	Keim	Does not asses factors
Comparative effectiveness study of flipped classroom versus online-only instruction of clinical reasoning for medical students	2023	Paul	Does not asses factors
"Flourish in the Clerkship Year": A Curriculum to Promote Wellbeing in Medical Students	2022	Flickinger	Does not asses factors
A Missed Case of Area Postrema Syndrome Presenting with Neuromyelitis Optica Spectrum Disorder	2022	Khan	Does not asses factors
Evaluating the Reliability and Validity Evidence of the RIME (Reporter-Interpreter-Manager-Educator) Framework for Summative Assessments Across Clerkships	2021	Ryan	Does not asses factors
Suicide Assessment and Management Team-Based Learning Module	2020	Lerchenfeldt	Does not asses factors
Medical and Surgical Education Challenges and Innovations in the COVID-19 Era: A Systematic Review	2020	Dedeilia	Does not asses factors
How to Teach Laboratory Stewardship in the Undergraduate Medical Curriculum?	2020	Roth	Does not asses factors
Missed opportunities in the way medical schools evaluate the ethical domain in clerkship rotations	2019	Dos Santos	Does not asses factors
Is there a role for GPs in teaching neurology to medical students? A qualitative evaluation.	2019	Jones	Does not asses factors
Using Instrument-Guided Team Reflection and Debriefing to Cultivate Teamwork Knowledge, Skills, and Attitudes in Pre-Clerkship Learning Teams	2019	Chen	Does not asses factors
Sleep and Epilepsy: A Complex Interplay	2017	Lanigar	Does not asses factors
Neurophobia: a chronic disease of medical students	2015	Solorzano	Does not asses factors
A standardized online clinical education and assessment tool for neurology clerkship students assigned to multiple sites	2014	Holland	Does not asses factors
Entrustable professional activities: A useful concept for neurology education	2018	Horak	Does not asses factors
A Case-Based Neuroanatomy Laboratory on the Neurobiology of Psychiatric Conditions for Second-Year Medical Students	2021	Porter-Stransky	Does not asses factors
Clerkship-Specific Medical Student Mistreatment	2018	Breed	Does not asses factors
The Clinical Microsystems Clerkship at the University of California, San Francisco: Integrating Clinical Skills and Health Systems Improvement for Early Medical Students	2023	Chang	Does not asses factors
Heart Rate Variability-Measured Stress and Academic Achievement in Medical Students	2021	Yoo	Does not asses factors
Exploring Empathy in the Face of Patient Anonymity and Professional Challenges and Barriers	2018	Mendoza	Does not asses factors
Integrating basic, clinical, and health system science in a medical neuroscience course of an integrated pre-clerkship curriculum	2023	Goodwin	Measuring performance outcomes other than NBME/Shelf scores
The relationship of online pre-recorded neurology mini-lectures to medical student assessment: a pilot study	2023	Benamer	Measuring performance outcomes other than NBME/Shelf scores
Medical student exam performance and perceptions of a COVID-19 pandemic-appropriate pre-clerkship medical physiology and pathophysiology curriculum	2022	Chang	Measuring performance outcomes other than NBME/Shelf scores
Educating Residents and Students in the Clinic	2023	Furr Stimming	Measuring performance outcomes other than NBME/Shelf scores
Components of Mid-clerkship Feedback in a Neurology Clerkship and Their Impact on Subsequent Student Performance	2022	Tarulli	Measuring performance outcomes other than NBME/Shelf scores
Predictors of faculty narrative evaluation quality in medical school clerkships	2022	Mooney	Measuring performance outcomes other than NBME/Shelf scores
"Flipped" clinical rotations: A novel approach	2022	Xiong	Measuring performance outcomes other than NBME/Shelf scores
Impact of a Hybrid-Virtual Teaching Model on the Physical Examination Skills of Fourth-Year Medical Students	2022	Zeldin	Measuring performance outcomes other than NBME/Shelf scores
Contemporary Neuroscience Core Curriculum for Medical Schools	2021	Gelb	Measuring performance outcomes other than NBME/Shelf scores
Education Research: A Qualitative Study on Student Perceptions of Neurology and Psychiatry Clerkship Integration	2021	Mowchun	Measuring performance outcomes other than NBME/Shelf scores
The role of residents in medical students' neurology education: current status and future perspectives	2020	Keser	Measuring performance outcomes other than NBME/Shelf scores
An observational study of an approach to accommodate a fourth-year to third-year neurology clerkship curricular transition	2020	Kraakevik	Measuring performance outcomes other than NBME/Shelf scores
A Neurology Clerkship Curriculum Using Video-Based Lectures and Just-in-Time Teaching (JiTT)	2018	Dominguez	Measuring performance outcomes other than NBME/Shelf scores
Imprinting on Clinical Rotations: Multisite Survey of High- and Low-Value Medical Student Behaviors and Relationship with Healthcare Intensity	2019	Leep Hunderfund	Measuring performance outcomes other than NBME/Shelf scores
An educational initiative to improve medical student awareness about brain death	2018	Lewis	Measuring performance outcomes other than NBME/Shelf scores
Incorporating students into the clinic may be associated with both improved clinical productivity and educational value	2017	Tanner	Measuring performance outcomes other than NBME/Shelf scores
Students with an autonomous role in hospital care - patients' perceptions	2018	Scheffer	Measuring performance outcomes other than NBME/Shelf scores
Education Research: Difficult conversations in neurology: Lessons learned from medical students	2018	Lemmon	Measuring performance outcomes other than NBME/Shelf scores
The effectiveness of TBL with real patients in neurology education in terms of knowledge retention, in-class engagement, and learner reactions	2017	Alimoglu	Measuring performance outcomes other than NBME/Shelf scores
Neurology objective structured clinical examination reliability using generalizability theory	2015	Blood	Measuring performance outcomes other than NBME/Shelf scores
Musculoskeletal Medicine Is Underrepresented in the American Medical School Clinical Curriculum	2016	DiGiovanni	Measuring performance outcomes other than NBME/Shelf scores
Structure of neuroscience clerkships in medical schools and matching in neuromedicine	2015	Albert	Measuring performance outcomes other than NBME/Shelf scores
A Brief Examination of Integrated Care in Undergraduate Medical Education	2015	Dub√©	Measuring performance outcomes other than NBME/Shelf scores
Workplace learning through peer groups in medical school clerkships	2014	Chou	Measuring performance outcomes other than NBME/Shelf scores
Evaluating team-based, lecture-based, and hybrid learning methods for neurology clerkship in China: a method-comparison study	2014	Yang	Measuring performance outcomes other than NBME/Shelf scores
The Impact of COVID-19 on Neurology Education: A Medical Student Perspective	2020	Guadix	Measuring performance outcomes other than NBME/Shelf scores
Optimizing the Use of an Online Self-assessment Exam to Promote Self-directed Learning Behaviors in Medical Students	2020	Ghosh	Measuring performance outcomes other than NBME/Shelf scores
Training in Neurology: Neuro Day: An Innovative Curriculum Connecting Medical Students With Patients	2021	Frey	Measuring performance outcomes other than NBME/Shelf scores
Evidence-oriented teaching of geriatric psychiatry: a narrative literature synthesis and pilot evaluation of a clerkship seminar	2022	Lenouvel	Measuring performance outcomes other than NBME/Shelf scores
Revisiting Feed Forward: Promoting a Student-Centered Approach to Education Handoffs, Remediation, and Clerkship Success	2023	Masangkay	Measuring performance outcomes other than NBME/Shelf scores
Developing a standardized EMR workflow for medical students and preceptors	2023	Viguera Altolaguirre	Measuring performance outcomes other than NBME/Shelf scores
Evaluating Teaching Effectiveness of Medical Humanities in an Integrated Clerkship Program by a Novel Prospective Propensity Score Matching Framework	2022	Chen	Measuring performance outcomes other than NBME/Shelf scores
Curricular response to COVID-19: real-time interactive telehealth experience (RITE) program	2021	Safdieh	Measuring performance outcomes other than NBME/Shelf scores
Transitioning preclinical students into clerkships amidst curricular disruptions from the COVID-19 pandemic	2021	Esquivel	Measuring performance outcomes other than NBME/Shelf scores
Instilling resiliency in surgical education: The benefits of longitudinal medical student learning	2021	Abel	Measuring performance outcomes other than NBME/Shelf scores
Identifying and Analyzing Systems Failures: An Interactive, Experiential Learning Approach to Quality Improvement for Clerkship-Level Medical Students	2021	Gheihman	Measuring performance outcomes other than NBME/Shelf scores
Expanding the duration of the neurology clerkship‚ does it matter?	2021	Keser	Measuring performance outcomes other than NBME/Shelf scores
An Interactive Approach to Teaching the Clinical Applications of Autonomy and Justice in the Context of Discharge Decision-Making	2020	Palanisamy	Measuring performance outcomes other than NBME/Shelf scores
Attracting neurology's next generation: A qualitative study of specialty choice and perceptions	2020	Jordan	Measuring performance outcomes other than NBME/Shelf scores
Continuity in Undergraduate Medical Education: Mission Not Accomplished	2019	Evans	Measuring performance outcomes other than NBME/Shelf scores
A quality improvement curriculum for the neurology clerkship: A practice-based approach to discharge education	2019	McInnis	Measuring performance outcomes other than NBME/Shelf scores
The Educational Climate Inventory: Measuring Students' Perceptions of the Preclerkship and Clerkship Settings	2017	Krupat	Measuring performance outcomes other than NBME/Shelf scores
Comprehensive opportunities for research and teaching experience (CORTEX): A mentorship program	2015	Zuzu√°rregui	Measuring performance outcomes other than NBME/Shelf scores
Using tablets to support self-regulated learning in a longitudinal integrated clerkship	2014	Alegr√≠a,	Measuring performance outcomes other than NBME/Shelf scores
Teaching patient-centered communication skills: a telephone follow-up curriculum for medical students	2014	Saba	Measuring performance outcomes other than NBME/Shelf scores
Status of neurology medical school education: Results of 2005 and 2012 clerkship director survey	2014	Carter	Measuring performance outcomes other than NBME/Shelf scores
Student views on the role of self-regulated learning in a surgery clerkship	2016	Lyons-Warren	Measuring performance outcomes other than NBME/Shelf scores
The value of neurology clerkship rotations at non-tertiary hospitals	2023	Villamar	No full text available
Does Delaying the United States Medical Licensing Examination Step 1 to After Clerkships Affect Student Performance on Clerkship Subject Examinations?	2021	Jurich	No full text available
Author response: Education Research: Positive effect of scheduled faculty modeling on clerkship student bedside skills exposure and learning	2017	Thompson Stone	No full text available
Novel dissection of the central nervous system to bridge gross anatomy and neuroscience for an integrated medical curriculum	2018	Hlavac	No full text available
Introduction to Quality Improvement and Systems-Based Practice: A Two-Part Module for Clinical Clerkship Students	2020	Gheihman	No full text available
Simulation-based education in neurology: lessons from the COVID-19 pandemic	2023	Toro	Studies of non-American medical schools
Influencing Factors of Future Specialty Choice for Undergraduate Medical Students: An Updated Experience from the UAE	2023	Al Zubaidi	Studies of non-American medical schools
Coproducing clinical curricula in undergraduate medical education: Student and faculty experiences in longitudinal integrated clerkships	2021	Gheihman	Studies of non-American medical schools
Career choice and influential factors among medical students majoring in psychiatry in China	2021	Zhang	Studies of non-American medical schools
How students and specialists appreciate the mini-clinical evaluation exercise (mini-CEX) in Indonesian clerkships	2020	Suhoyo	Studies of non-American medical schools
Influence of feedback characteristics on perceived learning value of feedback in clerkships: does culture matter?	2017	Suhoyo	Studies of non-American medical schools
Specialty Preferences and the Factors Influencing Them Among Pre-Clerkship Medical Students: The First Study from Alfaisal University-College of Medicine, Saudi Arabia	2016	Alsubaie	Studies of non-American medical schools
Student Continuity with Patients: A System Delivery Innovation to Benefit Patient Care and Learning (Continuity Patient Benefit)	2015	Poncelet	Studies of non-American medical schools
Learning Model to Achieve Clinical Reasoning Competency Using Technology-Enhanced Learning in Neurology Clinical Rotation: An Exploratory Study	2022	Nurhidayati	Studies of non-American medical schools
Clinical neuro-oncology formal education opportunities for medical students in the United States and Canada	2014	Dixit	Studies of non-American medical schools
Meeting international standards: A cultural approach in implementing the mini-CEX effectively in Indonesian clerkships	2014	Suhoyo	Studies of non-American medical schools
Generalizability of the Ottawa Surgical Competency Operating Room Evaluation (O-SCORE) Scale to Assess Medical Student Performance on Core EPAs in the Workplace: Findings From OneInstitution	2021	Ryan	Studies of non-American medical schools
The current state of headache medicine education in the United States and Canada: An observational, survey-based study of neurology clerkship directors and curriculum deans	2021	Pace	Studies of non-American medical schools
Clinical workplace learning: perceived learning value of individual and group feedback in a collectivistic culture	2018	Suhoyo	Studies of non-American medical schools
A multidisciplinary internship example in geriatrics	2014	Irem Budako«ßlu	Studies of non-American medical schools
Medical education trends for future physicians in the era of advanced technology and artificial intelligence: an integrative review	2019	Han	Systematic review
Interprofessional Education on the Neurology Clerkship for Physical Therapy and Medical Students	2023	Kung	Unrelated medical education study
Certification in Medical Simulation	2023	Whitworth	Unrelated medical education study
Longitudinal Mental Health Outcomes of Third-year Medical Students Rotating Through the Wards During COVID-19	2023	Stanislawski	Unrelated medical education study
Using the EPAs to Evaluate the Clinical Experience of Medical Students	2022	Bosinski	Unrelated medical education study
Evaluating Teaching Effectiveness of Medical Humanities in an Integrated Clerkship Program by a Novel Prospective Propensity Score Matching Framework	2022	Chen	Unrelated medical education study
Advanced Integrated Science Courses: Building a Skill Set to Engage With the Interface of Research and Medicine	2022	Miloslavsky	Unrelated medical education study
Clinically Isolated Brainstem Progressive Multifocal Leukoencephalopathy: Diagnostic Challenges	2022	Khan	Unrelated medical education study
How Do Clinical Electives during the Clerkship Year Influence Career Exploration? A Qualitative Study	2022	Sheu	Unrelated medical education study
The do's, don'ts and don't knows of establishing a¬†sustainable longitudinal integrated clerkship	2020	Bartlett	Unrelated medical education study
Education Research: The Narrative Evaluation Quality Instrument: Development of a tool to assess the assessor	2020	Kelly	Unrelated medical education study
Teaching Laboratory Stewardship in the Medical Student Core Clerkships Pathology-Teaches	2020	Roth	Unrelated medical education study
Core neurological examination items for neurology clerks: A modified Delphi study with a grass-roots approach	2018	Liu	Unrelated medical education study
Education in Pediatrics Across the Continuum (EPAC): First Steps Toward Realizing the Dream of Competency-Based Education	2018	Andrews	Unrelated medical education study
Tying knots: an activity theory analysis of student learning goals in clinical education	2017	Larsen	Unrelated medical education study
Child Neurology Recruitment and Training: Views of Residents and Child Neurologists From the 2015 AAP/CNS Workforce Survey	2017	Gilbert	Unrelated medical education study
High-Frequency Learning Goals: Using Self-Regulated Learning to Influence Day-to-Day Practice in Clinical Education	2017	Larsen	Unrelated medical education study
Opinion and Special Articles: Neurology education at US osteopathic medical schools	2017	Freedman	Unrelated medical education study
Evaluator Agreement in Medical Student Assessment Across a Multi-Campus Medical School During a Standardized Patient Encounter	2020	Braksick	Unrelated medical education study
Review of the Medical Student Performance Evaluation: analysis of the end-users‚ a perspective across the specialties	2021	Bird	Unrelated medical education study
Numerical versus narrative: A comparison between methods to measure medical student performance during clinical clerkships	2017	Bartels	Unrelated medical education study
Broadening learning communities during COVID-19: developing a curricular framework for telemedicine education in neurology	2021	Gummerson	Unrelated medical education study
Incorporating simulation technology into a neurology clerkship	2013	Ermak	Unrelated medical education study
Using a Mock Rounds Model and Neurology Patients to Teach Neurological Exam Skills in a Medical Neurobiology Course	2021	Lama	Unrelated medical education study
The demise of direct ophthalmoscopy: A modern clinical challenge	2015	MacKay	Unrelated medical education study
Moral distress and burnout in caring for older adults during medical school training	2020	Perni	Unrelated medical education study
The impact of medical students on work after clinic for neurology preceptors	2023	Breithaupt	Unrelated medical education study
Characterizing the Impact of Clinical Exposure to Patients with Opioid Use Disorder on Medical Students' Perceptions of Stigma and Patient Care	2023	Liu	Unrelated medical education study
Development of an assessment technique for basic science retention using the NBME subject exam data	2022	Matus	Unrelated medical education study
The Validity of MCAT Scores in Predicting Students' Performance and Progress in Medical School: Results From a Multisite Study	2022	Hanson	Unrelated medical education study
Expectations of and for Clerkship Directors 2.0: A Collaborative Statement from the Alliance for Clinical Education	2021	Morgenstern	Unrelated medical education study
Transforming the University of California, Irvine's medical physiology instruction into the pandemic era	2021	Lepe	Unrelated medical education study
Everyday Resilience: Equipping Faculty With Practical Exercises to Promote Resilience Among Medical Students	2021	Gheihman	Unrelated medical education study
Promoting Value Through Patient-Centered Communication: A Multisite Validity Study of Third-Year Medical Students	2020	Natt	Unrelated medical education study
Education Research: The medical student perspective on challenging conversations	2020	Willis	Unrelated medical education study
Embedding Ethics Education in Clinical Clerkships by Identifying Clinical Ethics Competencies: The Vanderbilt Experience	2020	Langerman	Unrelated medical education study
Characteristics of graduating US allopathic medical students pursuing a career in neurology	2019	Gutmann	Unrelated medical education study
Core curriculum guidelines for a required clinical neurology experience	2019	Safdieh	Unrelated medical education study
Faculty Development Revisited: A Systems-Based View of Stakeholder Development to Meet the Demands of Entrustable Professional Activity Implementation	2018	Lupi	Unrelated medical education study
Listening Beyond Auscultating: A Quality Initiative to Improve Communication Scores in the Hospital Consumer Assessment of Health Care Practitioners and Systems Survey	2018	Riegels	Unrelated medical education study
Emerging subspecialties in neurology: Sports neurology training and certification: An overview in 2018	2018	Morgenlander	Unrelated medical education study
Mistreatment of medical students in the third year may not be the problem	2017	Slavin	Unrelated medical education study
Effects of Mindfulness-Based Stress Reduction on the Mental Health of Clinical Clerkship Students: A Cluster-Randomized Controlled Trial	2017	van Dijk	Unrelated medical education study
The Community Preceptor Crisis: Recruiting and Retaining Community-Based Faculty to Teach Medical Students - A Shared Perspective From the Alliance for Clinical Education	2016	Christner	Unrelated medical education study
Changes in medical students' exposure to and attitudes about drug company interactions from 2003 to 2012: a multi-institutional follow-up survey	2015	Sierles	Unrelated medical education study
